# Method of Baking a Low-Fusing Inlay

**Published:** 1904-05

**Authors:** Alexander Jameson

**Affiliations:** Indianapolis, Ind.


### METHOD OF BAKING A LOW-FUSING INLAY.

Dr. Alexander Jameson, Indianapolis, Ind.

Take a cavity impression with "Archite;" mount it in a metal ring in a matrix of modeling compound. He places this ring in his swaging device and swages a small sheet or plate of pure gold over it. He takes away the resulting matrix and anneals it, then replaces on cavity impression, and puts a considerable quantity of moss-fiber gold upon it, and swages strongly. This gives him a matrix that is unshrinking and unchanging for all baking which he desires to do. With this matrix he bakes his low fusing body over a Bunsen burner. The advantages of this method are absolute accuracy of fit, minimum amount of time expended, and dispensing with a furnace.—Summary.

